# Mock circulatory loop applications for testing cardiovascular assist devices and *in vitro* studies

**DOI:** 10.3389/fphys.2023.1175919

**Published:** 2023-04-13

**Authors:** Ke-Wei Xu, Qi Gao, Min Wan, Ke Zhang

**Affiliations:** ^1^ Department of Engineering Mechanics, School of Aeronautics and Astronautics, Zhejiang University, Hangzhou, China; ^2^ Shandong Institute of Medical Device and Pharmaceutical Packaging Inspection, Jinan, China

**Keywords:** mock circulatory loop, cardiovascular assist device, mechanical circulation support, hemodynamics, in vitro experiment

## Abstract

The mock circulatory loop (MCL) is an *in vitro* experimental system that can provide continuous pulsatile flows and simulate different physiological or pathological parameters of the human circulation system. It is of great significance for testing cardiovascular assist device (CAD), which is a type of clinical instrument used to treat cardiovascular disease and alleviate the dilemma of insufficient donor hearts. The MCL installed with different types of CADs can simulate specific conditions of clinical surgery for evaluating the effectiveness and reliability of those CADs under the repeated performance tests and reliability tests. Also, patient-specific cardiovascular models can be employed in the circulation of MCL for targeted pathological study associated with hemodynamics. Therefore, The MCL system has various combinations of different functional units according to its richful applications, which are comprehensively reviewed in the current work. Four types of CADs including prosthetic heart valve (PHV), ventricular assist device (VAD), total artificial heart (TAH) and intra-aortic balloon pump (IABP) applied in MCL experiments are documented and compared in detail. Moreover, MCLs with more complicated structures for achieving advanced functions are further introduced, such as MCL for the pediatric application, MCL with anatomical phantoms and MCL synchronizing multiple circulation systems. By reviewing the constructions and functions of available MCLs, the features of MCLs for different applications are summarized, and directions of developing the MCLs are suggested.

## 1 Introduction

Cardiovascular disease has globally been a severe threat to human health and is a leading cause of death (Abubakar et al., 2015; Wang et al., 2016). According to an early report by the World Health Organization in 2008 (World Health Organization, 2008), the cause of the highest mortality in the world was cardiovascular disease. It showed that 7.2 million people died of ischemic heart disease and 5.7 million died of cerebrovascular disease in 2004. Recently, a Report from the American Heart Association in 2022 indicted that 19.05 million people worldwide died of cardiovascular disease, with an increase of 18.71% from 2010 (Tsao et al., 2022). Facing those severe heart diseases, heart transplantation might be a good treatment, especially for heart failure. However, there are very limited transplantable organs than needed (Rana et al., 2015; Westerdahl and Kobashigawa, 2019; NHS, 2020). As an alternative solution, artificial organs or long- and short-term cardiovascular assist devices (CADs) have been invented, such as ventricular assist device (VAD) (Givertz, 2011), total artificial heart (TAH) (Cook et al., 2015), prosthetic heart valve (PHV) (Musumeci et al., 2018), intra-aortic balloon pump (IABP) (Patterson et al., 2014; Khan and Siddiqui, 2022), etc. These CADs can be surgically implanted or percutaneously placed into the human body for artificially enhancing the cardiovascular circulation for a certain period. As an effective and widely used CAD, VAD can help to reduce the burden on the heart and maintain blood flow (Givertz, 2011), including left ventricular assist device (LVAD), right ventricular assist device (RVAD) and the biventricular assist device (BiVAD) (McDivit et al., 2014). VAD is usually a pump-type device that pumps the blood from the ventricle to the subsequent vessels. It can be a temporary surgical solution as a bridge to transplant, bridge to decision, or destination therapy in heart failure (HF) treatment (Ziemba and John, 2010; Gustafsson and Rogers, 2017). TAH is a mechanical assist device that replaces the human ventricles and valves. It is approved as a treatment for the end-stage biventricular HF and functions as a bridge to transplant (Cook et al., 2015). PHVs are normally applied to replace the native valves for some severe valvular heart diseases (Musumeci et al., 2018). The mechanical valve and the bioprosthetic valve are two typical PHVs (Pibarot and Dumesnil, 2009). IABP reduces afterload through counterpulsation as a way to increase cardiac output (CO) and treat the falling ventricles (Patterson et al., 2014; Khan and Siddiqui, 2022).

Ideally, CADs are expected to have excellent hemodynamic performance without any significant side-effects. Unfortunately, the side-effects are normally unavoidable, and some of them such as hemolysis and thrombosis would cause CAD malfunction, injury or even death to patients. Hence, it’s necessary to carry out tests on the reliability, safety and efficiency of CADs, which require repeated experimental validation before they are approved by the administrations and rolled out to the market (Orime et al., 1994; Clemente et al., 1997; Pantalos et al., 1998; Patel et al., 2005; Lee, 2009; Li S et al., 2020). The premarket experimental validation includes *in vitro* and *in vivo* experiments. *In vivo* experiments such as the acute and chronic tests on animals or patients provide the most valuable information about CAD working in the living body. However, the *in vivo* experiments are too expensive for an efficient iteration at the early stage of a product development. Contrarily, the *in vitro* experiments in a mock circulatory loop (MCL) system can be a cheaper and effective approach of intermediate validation for product development. MCL mimics the human cardiovascular system and allows CAD to be tested *in vitro*. Meanwhile, the *in vitro* experiments in MCL are easier to be controlled with high repeatability. Therefore, MCL plays an important role in the development of CAD at the design stage.

MCLs can be divided into three categories: mechanical mock circulatory loop (M-MCL), numerical mock circulatory loop (N-MCL) (Zhou et al., 1999; Schima et al., 2004; Wu et al., 2007; Geertsema et al., 2008) and hybrid mock circulatory loop (H-MCL) (Ferrari et al., 2001; Ferrari et al., 2002; Ferrari et al., 2003; Ferrari et al., 2005). M-MCL simulates CVS by means of hydraulic, mechanical and electrical components. N-MCL represents CVS by mathematical models. H-MCL is a combination of M-MCL and N-MCL. The current literatures show that most *in vitro* experimental researches focus on M-MCL. It can be directly applied to test different CADs under *in vitro* experiments, and also provide validation for N-MCL modeling. M-MCL is believed to be the fundamental for N-MCL and H-MCL. Hence, this article focuses only on the M-MCL and uses term ‘MCL’ to simply refer to ‘M-MCL’. As a powerful cardiovascular system *in vitro* simulator, MCL is able to mimic pulmonary circulation, coronary circulation, renal circulation, etc. Among the human physiological parameters, CO, heart rate, blood pressure, compliance and resistance are mostly concerned in designing MCL. Moreover, different types of CADs can be combined for joint testing under various physiological conditions (Khudzari et al., 2020). Therefore, MCL is multifunctional and suitable for application conditions of CADs, target patients and other purposes (Clemente et al., 1997; Pantalos et al., 1998; Lee, 2009).

One of the earliest MCLs, also called pulse duplicator, was invented by McMillan in the 1950s (McMillan et al., 1952; McMillan, 1955). It had basic ventricular components and was used to study heart valves motion in the pulse duplicator. After more than 70 years of development, MCL is now able to accommodate multiple components and circulations while simulating various physiological states. A comprehensive history of MCL development was documented by Khudzari et al. (2020). In addition, several researchers have reviewed MCL. For example, Cappon et al. (2021) presented the characteristics of three types of MCLs and introduced their development and advances. Shi et al. (2018) presented the structure, motion, and classification of MCLs (Shi and Yang, 2019). Baturalp and Ertas reviewed the advantages and disadvantages of the MCLs and gave their options for improvement (Baturalp and Ertas, 2015). In this article, we aimed to discuss MCLs based on their functional modules and their applications specifically for CAD developments and *in vitro* studies.

Besides the introduction section, the following parts of the article are divided into five sections. Section 2 presents an overview of the MCL structure. Section 3 introduces MCL applications based on different kinds of CADs, including PHV, VAD, TAH, and IABP. MCL with advanced structures of special functions and various circulation loops is introduced in Section 4. Section 5 provides a summary and suggestion of the MCL developments, followed with a conclusion in the last section.

## 2 Overview of MCL

**FIGURE 1:**
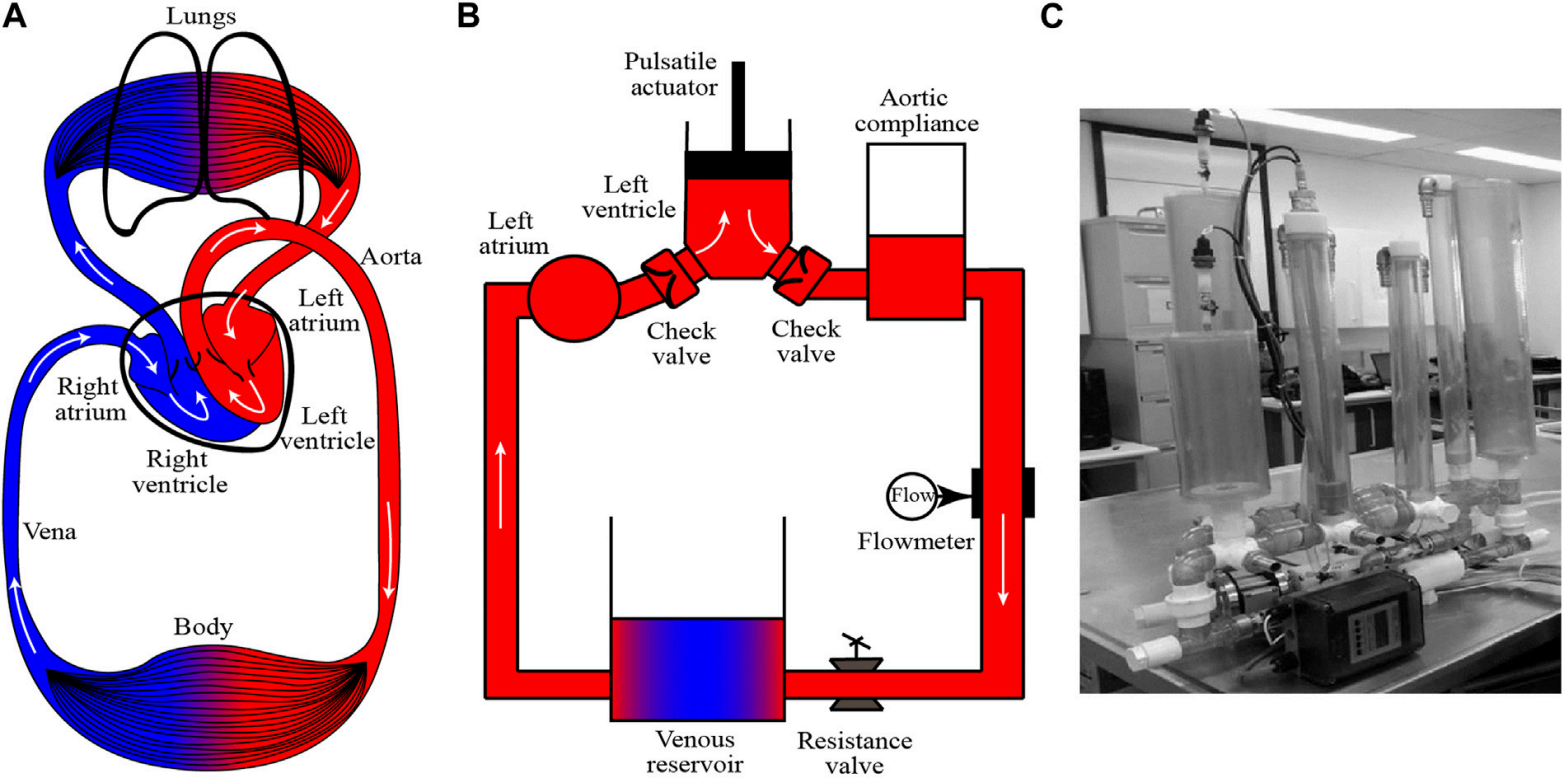
Illustration of **(A)** human cardiovascular system, **(B)** one basic MCL with systemic circulation and **(C)** one example of MCL from Mansouri et al. [Reproduced form (Mansouri et al., 2017)].

The human cardiovascular system (CVS) consists of the heart and blood vessels of the circulations. As shown in Figure 1A, CVS contains two basic circulations: the systemic circulation and the pulmonary circulation. The CVS can continuously provide blood for the human body, and can also quickly response to the change of physical status and adjust the blood flow (Rushmer, 1976). In CVS, the human heart is the most important organ which contains four chambers, namely, the left ventricle (LV), right ventricle (RV), left atrium (LA), and right atrium (RA). The basic function of the heart is to pump blood to the human body (Chandran et al., 2012). In the systemic circulation, blood is pumped out of LV and then flows to the aorta, peripheral blood vessels and RA in sequence. In the pulmonary circulation, blood is pumped from RV to the lung and LA.

To simulate CVS *in vitro*, MCL is invented. As shown in Figure 1B, a typical MCL of systemic circulation models the major organs in CVS and one photograph of MCL from Mansouri et al. (2017) is shown in Figure 1C. The component design and construction are important issues for the credibility of MCL. In order to improve the credibility of MCL, many researches have been conducted through simulating the Frank-Starling response (Gregory et al., 2011; Timms et al., 2011; Jansen-Park et al., 2016), optimizing the waveforms (Gregory et al., 2020; Jeong et al., 2020) and reducing the pressure loss (Gregory et al., 2010; Vandenberghe et al., 2011). In the MCL construction, the LV can be modeled by a rigid chamber driven by a linear motor (Tuzun et al., 2011; Ruiz et al., 2013; Bozkurt et al., 2016). The ventricle systole, diastole and heart beat can be simulated by the motor stroke and frequency. Additionally, the LV can be simulated by a pneumatically driven sac (Wu et al., 2004a; Pantalos et al., 2004; Wang Y et al., 2017). Compared with two driven approaches, the motor-driven method can easily, precisely and responsively control the circulation, while the pneumatic actuator can provide more realistic pressure supply. The aortic valve and mitral valve are normally modeled with industrial check valves or medical PHVs. The aortic compliance is essential for adjusting blood flow and pressure (Agrafiotis et al., 2021) in CVS, and it can be simulated by the Windkessel chamber, which is an airtight chamber with air and liquid. The peripheral resistance of the CVS can be simulated by a resistance valve or a throttle in the MCL. The vena is normally modularized by a reservoir. In some cases, the LA and vena can be simplified together as one reservoir. Common components of MCL are listed in Table 1 for comparing their functions and advantages. Besides, the water/glycerin mixture (35–40 weight percent glycerin), which has a similar density (1,060–1,100 kg/m^3^) and dynamic viscosity (3.5–3.6 cP) to the blood, is usually used as the working fluid for the *in vitro* experiment in the MCL (Triep et al., 2008; Nguyen et al., 2017; Gregory et al., 2020; Rozencwajg et al., 2022). For testing CADs and further applications, sophisticated MCLs are required to meet different applications, which will be further discussed in the following sections.

**TABLE 1:** Organs in the cardiovascular circulation correspond to major components in MCL and their advantages.

Organs in CVS	Models in MCL	Advantages
Ventricle	Rigid chamber with a linear motor (Tuzun et al., 2011; Ruiz et al., 2013; Bozkurt et al., 2016)	Easily controlled and with swift response
	Flexible sac driven pneumatically (Wu et al., 2004a; Pantalos et al., 2004; Wang Y et al., 2017)	More native pressure curve can be obtained from this model than that from rigid chamber model
Aorta	Airtight container with air above (Westerhof et al., 1971; Feng et al., 2017; Gehron et al., 2019)	This model is easy to be built and the compliance is simulated by the compressibility of air
	Spring capacitor (Ferrari et al., 1994; Woodruff et al., 1997; Pantalos et al., 2004)	The compliance is simulated by the elasticity of the spring instead of the air, thus this model consumes less volume than the airtight container. Meanwhile, the compliance can be adjusted by changing the load on the springs
	Flexible tubes (Pantalos et al., 2010; Ferrari et al., 2011; Gräf et al., 2015)	The compliance is simulated by the elasticity of the flexible tubes. By isolating the fluid and air, this model avoids the influence of air on the test fluid. This model is more attractive in the experiment with blood such as the *in vitro* hemolysis test
Valves	Check valve (Khienwad et al., 2019; Gregory et al., 2020; Li S et al., 2020)	Cheap and good reliability
	Prosthetic or bioprosthetic valves (Koenig et al., 2004; Schlöglhofer et al., 2013; Stanfield and Selzman, 2013)	These valves have less pressure loss and better hemodynamic performance than that of the check valves

## 3 Applications of MCL for CAD

### 3.1 Prosthetic heart valve

**FIGURE 2:**
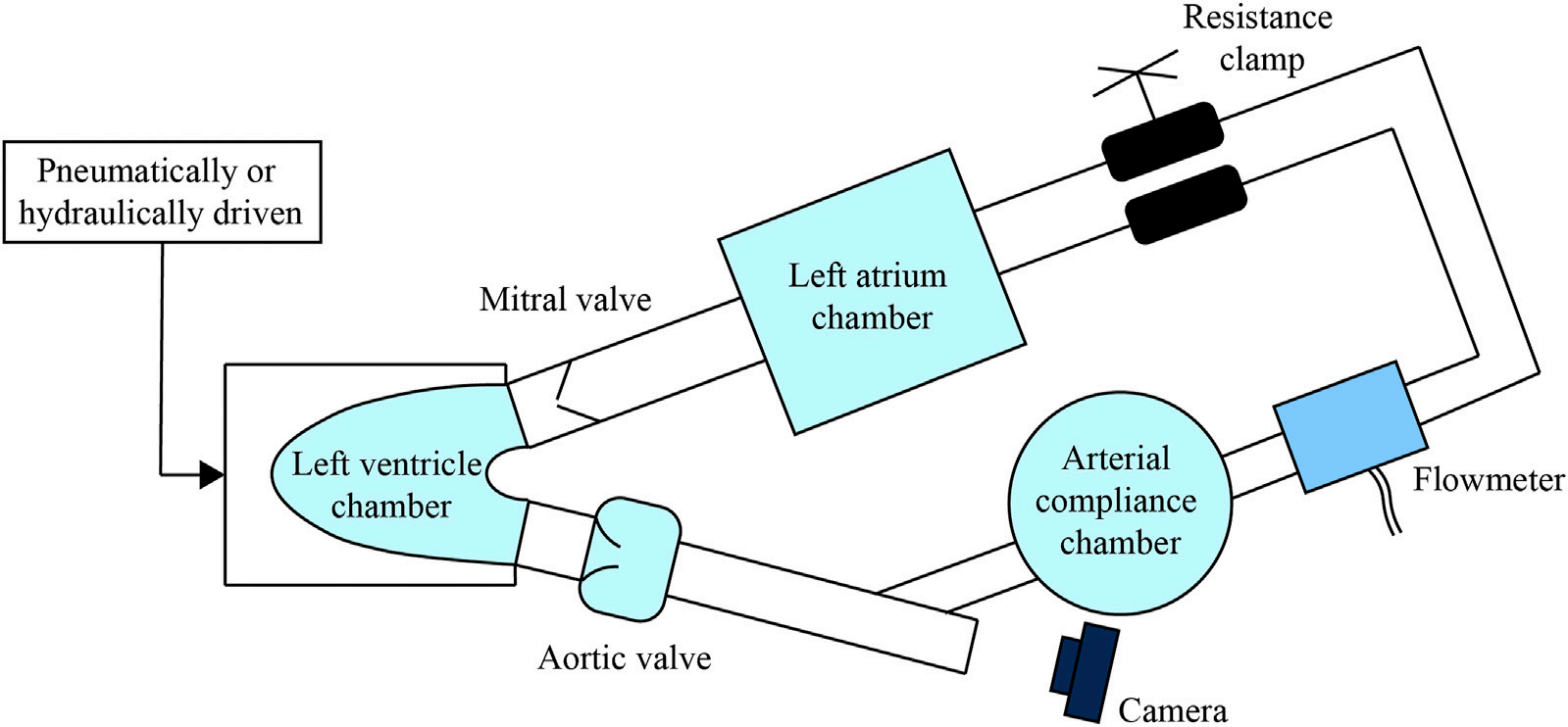
A simple MCL construction to study the PHV, make the aortic valve as an example to be tested. The branch-shaped visualized window and the camera can be used to observed the valve motion.

In 1950s, MCL applied to investigate the valves motion was also called pulse duplicator/simulator, which was driven in a relative simple manner (McMillan et al., 1952; Dávila et al., 1956; Raftery et al., 1968). For instance, the ventricular drive designed by Dávila et al. (1956) was composed of a motor, cam, rocker-arm, piston drive shaft, piston, and pump cylinder. This ventricular drive could supply the pulsatile pressure wave. In the recent decades, most MCLs used to study the PHV contain a flexible cavity, which is pneumatically (Fredrick Cornhill, 1977; Harasaki et al., 1979; Hartrumpf et al., 2003; Hildebrand et al., 2004; Suzuki et al., 2012; Schlöglhofer et al., 2013; Soucy et al., 2013; Tsuboko et al., 2016; Shehab et al., 2019; Hattori et al., 2021; Kado et al., 2021) or hydraulically (Heiliger et al., 1987; Suh et al., 1996; Linde et al., 2012; Feng et al., 2017; Nguyen et al., 2017) driven to simulate the ventricle beats. For example, air compressors or linear motors can be selected for driving the pneumatic or hydraulic components. Normally, the arterial compliance and left atrium chamber are necessary for an MCL to regulate the pressure waveforms. The peripheral resistance can be adjusted by the clamp. By this configuration, the valve hemodynamics and morphology can be studied by mounting the testing valve in the inlet or outlet of the MCL ventricle, as illustrated in Figure 2. In addition, some groups have built special dedicated platforms (Linde et al., 2012; Marcelli et al., 2018) to test valves, such as the vertical compact platform called THIA3 developed by Linde et al. (2012). It’s hydraulically driven by a linear motor with a piston at the conjunction section. All blood-contacting parts of the platform were made of blood-compatible materials. The platform could also be used to study the phenomenon of coagulation regarding the testing valve.

For more advanced MCLs, they can be designed with visual window and imaging system that enable researchers to straightforwardly study the motion and morphology of PHV, since improper opening and closing of the valve could lead to coagulation, reflux, and other consequences. What’s more, the morphology may cause valvular disease such as the aortopathy (Hattori et al., 2021). Thus, the morphology of native valves and PHVs are of great importance to study. MCL equipped with visual window is an effective solution to study the valve motion and morphology with the high-speed photography (Lin et al., 2000; Suzuki et al., 2012; Schlöglhofer et al., 2013; Tsuboko et al., 2016; Feng et al., 2017). For example, Suzuki et al. (2012) made an MCL that has a Y-shaped leaflets observation window between the valve chamber and the pulmonary arterial compliance chamber. Four kinds of PHVs with different leaflets were examined by the high-speed camera and echocardiography. This morphology study enabled them to design the optimal pulmonary valve. Similarly, an L-shaped configuration was built in the MCL (Feng et al., 2017) to observe the transcatheter aortic valve (TAV) motion by using the high-speed camera. The researches mentioned above reveal that the MCL embedded visualization technique is a powerful method to study the valvular morphology and guide the PHV design.

Besides the valve morphology, the hemodynamics is another essential issue of designing PHVs. Therefore, powerful experimental techniques are required for measuring flows around the testing valve so as to achieve a better understanding on the valve hemodynamics. Many researchers applied the particle image velocimetry (PIV) to capture the flow fields near the testing valves (Cenedese et al., 2005; Nguyen et al., 2017; Wang J et al., 2017). For example, Wang J et al. (2017) employed time-resolved PIV technique, combined with virtual dye visualization (VDV), finite-time Lyapunov exponents (FTLE), and other methods to study the internal flow field of an LV model with PHVs assembled. In their research, LV was a semi-ellipsoidal silicon sac. Although the configuration of their MCL without compliance was relatively simple, the measurement method did show the reliability and advantage of applying PIV on an MCL system for PHV study. In addition to an ideal semi-ellipsoidal silicon sac, the patient-specific ventricle silicon model can be used for more complicated applications. With the patient-specific silicon ventricle model based on computed tomography scanning, MCL can simulate the blood flow that is more similar to the native ventricle. Then, PIV technique can be applied to visualize the flow field inside the ventricle, and the flow information under the influence of PHV motion can be analyzed to guide the PHV design (Nguyen et al., 2017;Nguyen et al., 2019). Moreover, medical imaging equipment can also be used with MCL to study hemodynamics. For example, Hattori et al. (2021) adopted magnetic resonance imaging (MRI) to investigate the effect of bicuspid aortic valve (BAV) morphology on aortic jet flow quantitatively. They were the first to study the relationship between BAV morphology and aortic hemodynamics using the MRI compatible MCL. In conclusion, MCL combined with advanced measurement techniques enables deeper and better *in vitro* valvular hemodynamic investigations.

### 3.2 Ventricular assist device

**FIGURE 3:**
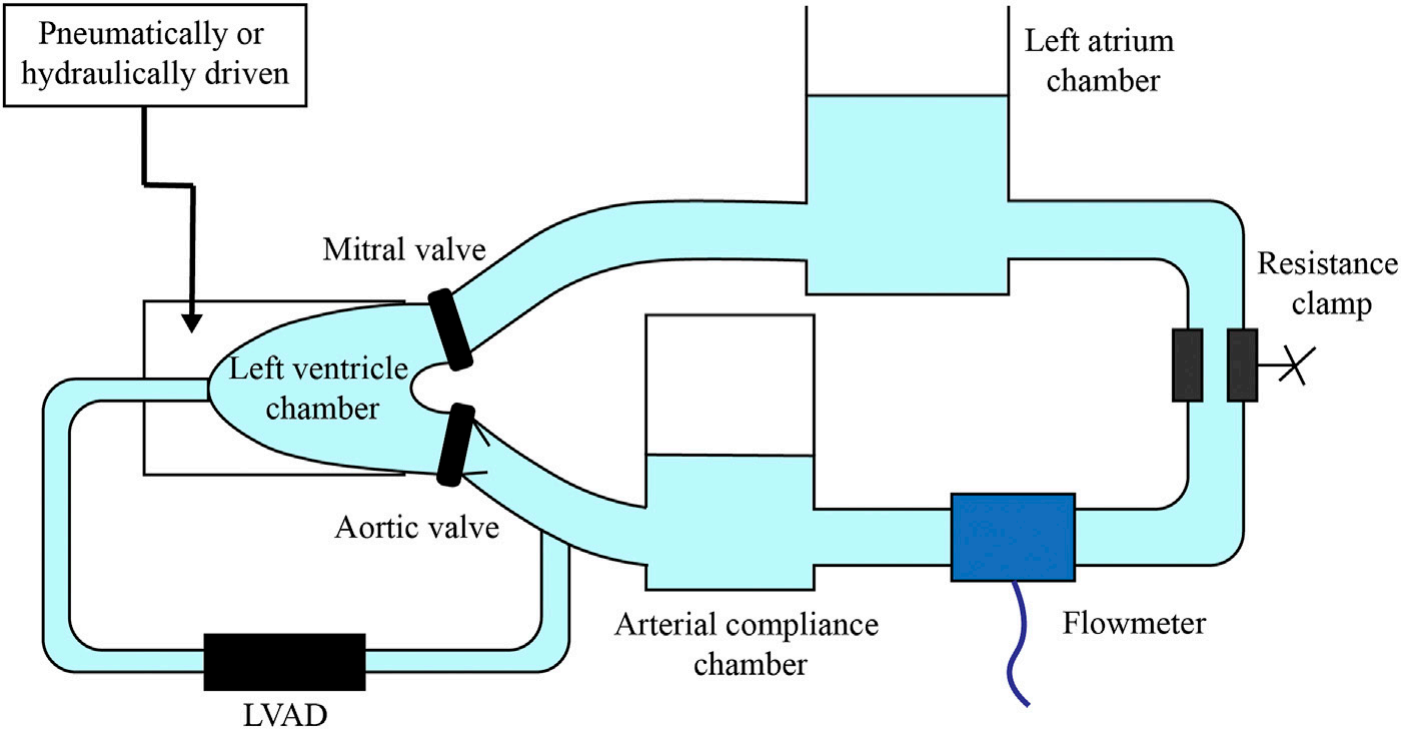
A simple MCL construction to study the VAD (take LVAD as an example).

Ventricular assist device is another medical instrument that is widely and normally used to help improve the CO of patients and treat the situation of heart failure. VADs are available in a variety of types and working modes to meet different surgical requirements. Therefore, the key to testing VADs with MCL is how to bring the VAD into the MCL platform and offer a proper working condition to simulate the related clinical ventricular circulation. To test the VAD (take the most commonly used LVAD as an example), the basic construction of most MCLs contains one left ventricle chamber, two valve models (aortic valve and mitral valve) and some Windkessel chambers (arterial compliance chamber and left atrium chamber) as shown in Figure 3. The ventricle chamber is usually made from flexible material and driven by pneumatic or hydraulic equipment. Since LVAD supports the failing heart by drawing blood from the left ventricle into the ascending aorta, the mock ventricle chamber has an opening at the apical end that connects the LVAD to the aortic behind the aortic valve. The opening in the apex of mock ventricle is the largest distinction between MCL specifically for the LVAD and other MCLs.

MCL can not only monitor and evaluate VADs, but also simulate diverse CVS working conditions, which are very important for widely testing the effectiveness and robustness of VADs. For the expensive *in vivo* experiments, they can only test one specific working condition in one case. However, with MCL, cheaper tests on the performance of VADs and their control strategies can be conducted in various CVS conditions. The LVAD performance tests are usually conducted *in vitro* before the *in vivo* tests, which is more dynamic and challenging (Snyder et al., 2022). Related studies tested the LVAD hydraulic performance in MCL for multiple operating conditions, and meanwhile *in vivo* tests were conducted for specific operating conditions to evaluate the overall performance (Oh et al., 2005; Jahanmir et al., 2008; Murakami et al., 2008). Although there are some differences between the results obtained from MCL and those obtained *in vivo*, such as the pressure waveforms variations, the findings of those studies demonstrate that *in vitro* experiments are essential for confirming LVAD performance. Moreover, the MCLs would become more powerful if they can be installed with optical windows, integrated with various sensors and combined with multiple modules. These features will greatly help MCL to test the fundamental hydrodynamic performance and the control strategies for VADs, to verify its hemocompatibility, and to evaluate the effects of VADs on CVS and organs. These three aspects will be discussed in the following subsections from 3.2.1 to 3.2.3.

#### 3.2.1 Test of fundamental hydrodynamic performance and control strategy

The MCL serves as a reliable platform to examine whether the hydrodynamic performance of VAD can meet its design requirement. The most fundamental parameters of a VAD, such as rotational speed, flow rate and pressure, can significantly affect the effectiveness of VAD support. For example, adequate pressure and flow rate can overcome regurgitation as well as obtain enough perfusion, but excessive flow can reduce aortic valve opening time and bring some complications (Tuzun et al., 2011). Therefore, hydrodynamic performance testing of VAD in a physiologic-like environment is needed to analyze the relationships of pressure, flow rate, rotation speed, power and so on for the in-depth evaluation and further development of VADs (Goldstein et al., 1992; Bearnson et al., 1996; Iijima et al., 1997; Yoshino et al., 2001; Grigioni et al., 2003; Vandenberghe et al., 2003; Oh et al., 2005; Miller et al., 2008; Murakami et al., 2008; Stanfield and Selzman, 2012; Rungsirigulnan et al., 2013). For instance, Tuzun et al. (2011) tested LVAD in an MCL driven by a piston pump. By adjusting the LVAD operation rotation speed, outflow resistance and stroke volume, they measured the LV pressure, aortic pressure and flowrate to validate the hydrodynamic performance of the tested LVAD. Meanwhile, the pressure influence on the aortic valve was evaluated. This research is a typical application showing that MCL can be successfully combined with other medical devices like VADs for testing their hydrodynamic performance *in vitro*.

Hydrodynamic performance is the most basic function of VADs. The research of hydrodynamic performance is mainly to understand the operation of VADs under complex conditions, so as to design and optimize the control of VADs. VADs, especially some long-term VADs, need optimized control strategies to cope with different physiological conditions, normally rest, sleep, exercise and gradual ventricular recovery after implantation, or even to avoid ventricular collapse caused by VADs. In addition, some pulsatile VADs need to be controlled and synchronized with the cardiac cycle to reduce blood clots, minimize energy expenditure, etc. These applications require VAD to have appropriate and optimized control strategies under complex working conditions. By simulating a wide range of physiological conditions, MCLs enable researchers to test and develop VAD control strategies (Yoshizawa et al., 1992; Williams et al., 1994; Ferrari et al., 1996; Kikugawa et al., 2001; Wu et al., 2004a; Gwak et al., 2005; Wu et al., 2020) to achieve optimal performance under different physiological conditions. Take the MCL designed by Wu et al. (2004a) as an example (Wu et al., 2007), the MCL owns systemic and pulmonary circulations. It consists of a semi-ellipsoidal silicone LV placed in a sealed air chamber driven pneumatically. An LVAD is connected from the ventricular chamber apical to the aortic loop behind the aortic valve. The LVAD was tested on the MCL under several pathological conditions, and the controller was improved to have an optimized effect. The result indicates that applying MCL to optimize VAD hydrodynamic performance and its control strategy is an effective method.

Although a wide range of physiological conditions can be simulated, MCL is still not able to fully mimic the native blood circulation. The limitations of MCL should also be concerned. For example, the Frank-Starling response is lacked in some MCL platforms. Frank-Starling response is an adaptive mechanism that the heart regulate its contractive force according to the filling volume in the ventricle. (Gregory et al., 2011; Sequeira and van der Velden, 2015; Gregory et al., 2020). Without the Frank-Starling Response, the LVAD test results would differ from those *in vivo*, especially regarding to the pressure and CO. In general, the MCL can macroscopically simulate the hemodynamic quantities very well, while it has some difficulties on providing phase information or fitting the waveforms of human circulation precisely. The issues that arises in Wu’s platform (Wu et al., 2004a; Wu et al., 2004b; Wu et al., 2007) are common problems that exist in many MCLs, which shows that MCL cannot completely replicate the human physiological environment and may affect the test results of CAD studies. Therefore, people have to face these problems when they are developing and optimizing MCLs.

#### 3.2.2 Assessment of hemocompatibility

The MCL can allow blood to flow in a physiological environment, where the effect of VAD on hemocompatibility can be studied *in vitro*. When a working VAD strongly interacts with blood, the shear flow generated by high-speed rotation of the VAD can cause trauma to red blood cells, resulting in adverse consequences such as shear induced hemolysis and thrombus. Therefore, the hemocompatibility test of the VAD in the MCL is necessary. Numerous researchers chose MCLs with constant flow to perform the VAD hemocompatibility test, such as the hemolytic (Cahill and Kolff, 1959; Takami et al., 1996; Rungsirikunnan et al., 2018) and thrombotic (Linneweber et al., 2002; Linneweber et al., 2008; Maruyama et al., 2009; Dassanayaka et al., 2013; Zayat et al., 2019) investigations. For instance, a test loop was recommended by American Society of Testing Materials (ASTM) to conduct the *in vitro* hemolysis test on continuous VAD (ASTM F1841-19, 2019). In this standard, the blood for the *in vitro* hemolysis test is generally less than 500 mL and maintained at the target temperature by water bath or heat exchanger. The VAD, the reservoir, the flowmeter, the clamp and several measurement ports are required in the test loop. There are also platforms designed specifically for the pulsatile VAD, such as the MCL built by Brockhaus et al. (2021) for testing thrombus generation in the pulsatile condition. In their study, the MCL has a compliance chamber, and all blood contact materials are biocompatible silicone. Blood does not come into contact with air, and there is a filter in front of the VAD inlet to prevent micro thrombi from entering the test equipment. It follows that the MCL for testing the hemocompatibility of the VAD has requirements for materials and airtightness. The blood-contact materials of the MCL components should be biocompatible. Also, the design of the compliance chamber needs optimization so that blood and air cannot be in direct contact. Meanwhile the MCL needs to be able to maintain a certain blood temperature.

Compared with clinical testing, simulation on dynamic balance of blood indices, monitoring multiple hematologic/biochemical data, and performing long-term hemocompatibility testing are still difficult to be perfectly achieved *in vitro* experiments with MCLs (Chen et al., 2011; Nix et al., 2020). These issues are unavoidable limitations to *in vitro* tests. However, MCL-based *in vitro* hemocompatibility assessment aims to solve the most prominent problems (e.g., thrombus and hemolysis caused by CAD) at a relatively low cost and expense. Therefore *in vitro* hemocompatibility assessment is of great value for rapid development and reliability improvement of VADs.

#### 3.2.3 Study on influence of VAD to CVS

In addition to simulating physiological conditions for the tests of VAD performance, the MCL can also assess the effect of the VAD on the entire circulatory system or related organs. This is necessary because the presence of the VAD can directly cause some changes of the circulatory system, such as the impact on blood pressure, ventricular pressure volume (PV) relations and nearby organs. Under some specific conditions, the VAD may even cause ventricular collapse and significant aortic valve insufficiency. Studying and testing the influence of the VAD on the CVS is crucial to ensure the reliability and safety of VAD products. Therefore, in order to evaluate the interaction between the VAD and the human circulatory system (Giridharan et al., 2004; Jansen-Park et al., 2016; Zhuang et al., 2016; Wang Y et al., 2017; Khienwad et al., 2019) and to assess the effects of VADs on nearby organs such as aortic valve (Gardner et al., 1993; Tuzun et al., 2011) and left ventricle (Gwak et al., 2005; Voigt et al., 2005), MCLs were developed for *in vitro* tests of VADs. Akiyama et al. (2018) performed a typical investigation on aortic insufficiency resulting from a long-term LVAD. In their work, aortic valve insufficiency was simulated by a bypass loop in parallel with the aortic valve. Compared with the conventional qualitative ultrasound evaluation of aortic insufficiency, this research proposed a quantitative thermodilution method to measure the shunt caused by aortic insufficiency. Their research also indicated that various techniques can be applied to the MCL experiments. More and more studies have suggested that the MCL plays an important role in testing the potential risks of VAD to patients.

#### 3.2.4 MCL specifically for RVAD/BiVAD/pVAD test

Although a large proportion of VADs are LVADs, there are also a certain number of RVADs and BiVADs available in the market. The MCL can also be used in studies of these types of VADs. The difference is that MCLs built for the study of RVAD or BiVAD usually require both systemic and pulmonary circulations (Kresh et al., 1990; Takami et al., 1996; Stevens et al., 2014; Mueller et al., 2017; Shehab et al., 2017; Stephens et al., 2017; Huang and Zhu, 2019; Kado et al., 2020). Kado et al. (2020) designed an MCL with both systemic and pulmonary circulations, driven by two pneumatic pumps functioning as two ventricles, for the evaluation of biventricular devices. This MCL has optional ports for atrial and ventricular cannulation while simulating the physiological conditions of healthy CVS, left HF and right HF. Although two circulatory systems need to be simulated, the convenient modular design of the MCL makes it easy to cope with different requirements.

Over the last decade, percutaneous ventricular assist device (pVAD), such as the Impella series (Abiomed Inc., MA, United States), has been developed and widely used. Unlike the traditional LVAD, the pVAD placement does not require an opening operation in the apex of the ventricle and can be percutaneously inserted from the femoral or brachial artery, with the entrance through the heart valve into the ventricle. The pVAD pumps blood from the ventricle to the aorta and increases the CO. Due to the unique intervention mode of the pVAD, the suitable MCL does not have an opening designed at the apex of the mock ventricle, but a percutaneous access is required at the femoral or axillary artery. Thanks to the minimally invasive surgical approach and effective clinical performance of the pVAD, an increasing number of studies have been carried on the pVAD. Some researchers have built continuous flow MCLs to conduct performance tests and hemolysis tests on pVADs (Triep et al., 2006a; Triep et al., 2006b; Roberts et al., 2020), while pulsatile MCLs for testing pVADs can be found in literatures (Harasaki et al., 1979; Wang Y et al., 2017; Chang et al., 2018). Among the researches, Chang et al. (2018) installed the pVAD between the ventricular chamber and the aortic chamber in their MCL system. This bypass structure makes an independent measurement of the pVAD possible. In the MCL of Wang Y et al. (2017), the left ventricle was not a simplified mechanical component but a pneumatic silicon sac. The pVAD was directly placed into the left ventricle from the ascending aorta through the aortic valve. Their MCL design with ascending aorta model is very important for testing the implantation of pVAD and investigating the interaction between the pVAD and the silicon models of the CVS. Various applications of the MCLs to different types of VADs have demonstrated that MCLs can flexibly assemble powerful functional modules based on different requirements. Furthermore, specifically designed MCLs can be easily controlled to simulate complicated physiological conditions.

### 3.3 Total artificial heart

Different from VAD, TAH is a kind of CAD that replaces native ventricles and valves of patients (Cook et al., 2015). Due to its special feature, the MCL used to test the TAH does not inherently have a power source. The testing TAH actually acts as the power source of the MCL, and its capability of driving circulation becomes the primary research content. When the TAH is implanted into the human CVS, it replaces the patient’s two ventricles and artificially achieves the systemic and pulmonary circulation. Therefore, the MCL must contain both systemic and pulmonary circulation loops. The TAH assembled within an MCL system is normally tested for performance evaluation (Castañeda et al., 1964; Kito et al., 1976; duPlessis and Tsang, 1977; Shiose et al., 2010; Pelletier et al., 2015; Pelletier et al., 2016; Tozzi et al., 2017), control strategy optimization (Wang et al., 1987; Nabel et al., 1990), malfunction evaluation (Makino et al., 2006), and durability test (Zapanta et al., 2005) prior to animal experiment or clinical test. In the design of the MCL targeting TAH tests, a series of achievements were formed (Donovan, 1975; Minamitani et al., 1978; High et al., 1979; Imachi et al., 1992a; Imachi et al., 1992b; Khalil et al., 2008; Fukamachi et al., 2010). The earliest MCL used to study the artificial heart was designed by Kolff, (1959), and the resistance cannot be arbitrarily controlled in the loop. One widely used MCL for testing TAH is called Donovan Mock Circulation System (DMCS) (Donovan, 1975). The entire DMCS is a tank designed with internal systemic venous compliance (SVC), aortic compliance (AoC), pulmonary arterial compliance (PAC) and pulmonary venous compliance (PVC) chambers, with the compliance of the four chambers determined by the volume of air above the chambers. The systemic and pulmonary resistance are achieved by bellows-operated valves. Each of the four chambers has one port for connection to the TAH. Many researchers applied DMCS to the performance tests of TAHs (Nabel et al., 1990; Vonesh et al., 1991; Sueshiro et al., 1997; Sueshiro et al., 1998; Olegario et al., 2003; Crosby et al., 2015; Crosby et al., 2017). Olegario et al. (2003) proposed a control strategy to prevent the atrial suction of a continuous flow TAH (CFTAH), tested with two centrifugal blood pumps and DMCS. Crosby et al. (2015) calibrated the PV relationship of SynCardia TAH (SynCardia, Systems Tucson, AZ, United States) by varying the preload and afterload in DMCS. Fukamachi et al. (2010) conducted a series of studies on TAHs using their MCL, which is also compatible with single-ventricle VAD. Their system includes both systemic and pulmonary circulations for the testing of their CFTAH (Fukamachi et al., 2010; Shiose et al., 2010), pediatric CFTAH (Fukamachi et al., 2018), and virtual MCL platforms (Miyamoto et al., 2019).

Most of the studies are mainly focused on the testing of the TAH performance, but few researchers applied hemocompatibility tests at the MCL. Different from the platform suggested by ASTM for LVAD (ASTM F1841-19, 2019), the compliance chamber is necessary for the TAH *in vitro* hemolysis test. For example, Orime et al. (1994) presented an MCL for the TAH *in vitro* hemolysis, with a system owning flexible reservoir and compliance tube. Gräf et al. (2015) developed an MCL for the *in vitro* hemolysis test. The interior of their MCL was made of hemo-compatible material, and the blood does not come into contact with the air. The silicone tube inside the arterial compliance chamber separates the blood from the air, which has the effect of isolating the air while simulating compliance. It is believed that more MCL experiments will consider hemocompatibility in the future, and hemocompatibility will become a major concern.

### 3.4 Intra-aortic balloon pump

The IABP is a short-term assist device, of which the long intra-aortic balloon (IAB) is placed in the aorta and provides perfusion to the heart through its contraction and expansion. The IABP can be inserted percutaneously and guided into the aorta to perform a counterpulsation pumping. During heart diastole, the IAB inflates, and pushes more blood flow to the coronary arteries. During heart systole, the IAB deflates, and enables more blood flow to the body (Patterson et al., 2014; Khan and Siddiqui, 2022).

The specificity of the MCL for testing the IABP lies in the need for an aorta resembling component with the IABP insertion (e.g., aorta model) as well as a device for synchronizing the IABP. Typically, researchers design a simple structure such as a piece of pipe in the MCL for the IABP placement (Wolf and Clinch, 1972; Niederer and Schilt, 1988; Sakamoto et al., 1996; Papaioannou et al., 2004; Schampaert et al., 2011; Farag et al., 2022). Moreover, in order to simulate the clinical placement process more realistically, the aortic trunk model can be included to the MCL (Imanishi et al., 1989; Biglino et al., 2012b; Kolyva et al., 2012). In response to synchronization with the IABP, the IABP can be triggered by an MCL-simulated electrocardiographic signals (Schampaert et al., 2011), by pressure signals from the ventricles (Farag et al., 2022), or synchronously by ventricular driver signals (Wieting et al., 1971; Niederer and Schilt, 1988; Papaioannou et al., 2004; Biglino et al., 2012b; Kolyva et al., 2012). For example, Schampaert et al. (2011) mimicked the aorta using a cylindrical polyurethane tube with the diameter close to the human aorta. The MCL contains systemic, pulmonary and coronary circulation, with the two ventricles driven by two piston pumps. The IABP was inserted through the artificial side branch of the aorta and triggered by an MCL-simulated electrocardiogram (ECG) signal at the onset of ventricle systole. This simple structure is suitable for the basic IABP performance test. In terms of more advanced structures, the MCL design of Kolyva et al. (2012) utilized an anatomically based real-size arterial model with an IABP inserted from the femoral artery. The pulsatile blood flow was provided through a VAD with dual chambers of the left ventricle and atrium. The branches of arterial model are ended with capillaries to simulate the resistance, and the compliance is achieved through injectors. Similarly, Biglino’s experimental platform also has an aortic model based on anatomical dimensions, and the MCL can be adjusted at multiple angles to simulate semirecumbent positions (Biglino et al., 2012b). The ventricular model is a LVAD, while the IABP was synchronized and triggered at the beginning of diastole as counterpulsation. These MCLs with anatomical models allowed investigators to simulate the insertion process of the IABP and simultaneously test the IABP performance. This functionality of the MCL also illustrates the powerful ability of the MCL to perform performance test and simulate surgical procedures.

## 4 Advanced MCL with complex circulations for other *in vitro* studies

### 4.1 Pediatric MCL

Most of the CADs are designed for adults, and the corresponding MCLs are built according to the physiological characteristics of adults as well. There are fewer CADs and MCLs for neonatal and pediatric patients. However, a certain number of children do suffer from cardiovascular diseases and need the CAD assistance for the surgical treatment. Therefore, it is essential to build MCLs based on physiological parameters specifically for testing CADs of pediatric usage. The circulatory system of pediatric patients is normally the same as that of adults, but some hemodynamic characteristics are different. For example, the heart rate of pediatric patients is generally higher than that of adults, the CO is smaller, and the aortic pressure is lower. For these considerations, researchers made multiple solutions of the MCL specifically for pediatric usage (Pantalos et al., 2009; Vandenberghe et al., 2011; Huang et al., 2013; Telyshev et al., 2017; Tozzi et al., 2017; Fukamachi et al., 2018; Torres et al., 2021). The structures of pediatric MCLs used to test for CADs are similar with the adult MCLs. They include basic component such as LV, systemic aortic compliance (SAC) and systemic vascular resistance (SVR). The pediatric MCL built by Pantalos et al. (2010) is applicable to a variety of simulations and studies. It has both left and right ventricles with atriums as well, and enables to test the IABP, the LVAD, the extracorporeal membrane oxygenation (ECMO) and other types of CADs. The neonatal MCL developed by Trittenwein et al. (1998) was used to test the neonatal ECMO, driven by a roller pump, with a whole circuit volume of 200 mL, which is equivalent to the blood volume of a 2.7 kg infant (Trittenwein et al., 1999; Trittenwein et al., 2001). Apparently, these pediatric MCLs can be modified directly from the adult MCL, so the CAD can be tested under standard running conditions of pediatric patients.

More importantly, in addition to testing pediatric CADs, another concern for pediatric MCL is the simulation of surgical procedures, especially the procedures for treating congenital heart disease (CHD). One of the optimal periods for a CHD surgery is in the pediatric age, such as Fontan surgery usually performed around 2–3 years old (Yamada et al., 2013). Before a clinical surgery, the simulation of surgical process in the MCL enables the doctors to assess the outcome and hemodynamic performance of surgery procedures for a specific case. Different from the MCL for the CAD evaluation, the MCL used to evaluate surgery often mimics the pediatric physiologic anatomy of the patient with CHD (Biglino et al., 2012a; Zhou et al., 2015; Hang et al., 2016). Hang et al. (2016) designed an MCL that contains chambers to mimic the left common carotid artery compliance, left subclavian artery compliance, shunt and upper body resistance, etc. Several patient-specific aortic arch models were connected to the MCL for experiments. This kind of powerful MCL was expected to conduct further physiology and morphology researches.

Since the types of pediatric CHD are very complex, the related surgical procedures are also relatively complicated. Therefore, MCL simulations for specific surgical procedures have become valuable to study. The hypoplastic left heart syndrome (HLHS) is a type of CHD that the left ventricle is undeveloped or absent (Biglino et al., 2012a; Hang et al., 2016). The main surgical procedures for HLHS include the Norwood procedure, bidirectional Glenn procedure, and the subsequent Fontan procedure. Various investigations have been performed on pediatric MCLs assembled with aortic models to evaluate the hemodynamic performance of these procedures (Biglino et al., 2011; Biglino et al., 2012a; Zhou et al., 2015; Hang et al., 2016). Among the surgical procedures, considerable researches have focused on the simulation of the Fontan procedure (Haggerty et al., 2012; Giridharan et al., 2013; Kerlo et al., 2013; Vukicevic et al., 2013; Giridharan et al., 2014; Horvath et al., 2020), which has greatly improved patient survival and is the most effective treatment (Kutty et al., 2020). Depending on the physiological features of the Fontan patient, the MCL has a single ventricle driving the whole MCL. The ventricle can be pneumatically or hydraulically driven with a silicone chamber (Rodefeld et al., 2010; Giridharan et al., 2013; Giridharan et al., 2014) or a VAD (Vukicevic et al., 2013). Other components of Fontan -MCL normally include AOC, SVR, SVC, PAC, and pulmonary vascular resistance (PVR). To simulate the univentricular Fontan circulation, it should also integrate the cavopulmonary junction model. Horvath et al. (2020) designed a powerful MCL based on the complex anatomical structure of the patient. Their MCL has a diaphragm separating the thoracic and abdominal cavities with corresponding pulmonary compliance and abdominal compliance. The venous flow is a combination of the SVC flow and the inferior vena cava (IVC) flow. When the pneumatic artificial muscle actuators push and pull the diaphragm, the volume of the abdominal and the chest cavities change, and the pressure in the corresponding cavity also changes to produce a pressure gradient. The pressure gradient accelerates the fluid. This complex MCL system takes into account the influence of thoracoabdominal compliance and respiratory pressure, thus enabling more credible experimental results to be obtained. The applications of MCLs in pediatric CHD are well indicated that a more realistic simulation of the characteristics of the pathology and surgical protocol in the MCL can improve surgical outcomes.

### 4.2 MCL combined with anatomical phantoms

In order to simulate the CVS better as well as more deeply investigate the hemodynamics inside of the organs, the anatomical phantoms or patient-specific models can be introduced into MCLs. Replacing the flexible silicone sacs with patient-specific anatomical ventricle phantoms can provide a more realistic simulation of the human body, which is a common strategy for testing PHV and VAD with MCL. By using those transparent phantoms, MCL can work under methods of flow visualization (Cassot et al., 1985), PIV (Tanné et al., 2010; Nguyen et al., 2017; Nguyen et al., 2019) and other measurement techniques to perform internal hemodynamic investigations.

On the one hand, MCLs combined with anatomical phantoms benefit the CAD research and design. The physical information inside the anatomical phantoms, such as velocity and shear stress, provides inspirations for the CAD design and optimization. Nguyen et al. (2017) built the MCL incorporating a patient-specific right ventricle phantom to investigate the hemodynamics under the influence of the valves (Nguyen et al., 2019). Their findings gave clues to the heart valve improvement. On the other hand, anatomical phantoms combined with MCL are able to help investigate the cause of cardiovascular pathology. For some arterial lesion problems, researches that involve arterial models or patient-specific arterial models to the MCL system can help simulate blood flow phenomena, understand the lesion pathology and guide treatment options (Klanchar et al., 1990; Knoops et al., 2017; Bonfanti et al., 2020; Gehron et al., 2020; Li X et al., 2020). For example, Zhou et al. (2015) designed an MCL coupled with three models to compare the hemodynamic performance of three types of surgical procedures. Their result showed the potential of a new surgery. This is quite a representative application of incorporating a partial vascular model into the MCL for targeted research. Some other MCL platforms for multipurpose studies would access more complex models. The MCL built by Gehron et al. (2019) has a normal human-sized arterial and venous phantoms, with the pneumatic ventricle as the power source. All arterial branches have combined peripheral resistance and compliance lumen. The platform can be combined with different measurement techniques for fluid mechanics studies without the biologic constraints. Similarly, the MCL built by Li X et al. (2020) connects a phantom of patient-specific aorta with 12 branches. The MCL is multifunctional for studying the hemodynamics of aortic dissection, evaluating CADs and providing a training platform for interventional treatment. It can be seen that MCLs with anatomical phantoms can serve a wide range of reproducible experiments *in vitro*. Moreover, phantoms with transparent materials can broaden the range of test methods, enabling, for example, optical methods to directly measure the internal flow of phantoms *in vitro*.

### 4.3 MCL with more circulations

The human circulatory system is complex while researchers commonly focus on the systemic and pulmonary circulations, which are basic and relatively easy to implement on the MCL. However, when more circulations are concerned, or when the operation of some medical devices affects different circulations, more circulatory loops are required to be coupled to the MCL. Since coronary artery disease accounts for a large proportion of cardiovascular disease, many scholars have incorporated coronary circulation in the MCL (Geven et al., 2004; Koenig et al., 2004; Pantalos et al., 2004; Park et al., 2006; Kaebnick et al., 2007; Park et al., 2007; Zannoli et al., 2009; Schampaert et al., 2011; Giridharan et al., 2012; Schampaert et al., 2014; Madukauwa-David et al., 2020). The flow characteristics of coronary circulation can be realized by means of the stepping motor cooperating with the valve (Calderan et al., 2016), the parallel pipeline with the solenoid valve (Rezaienia et al., 2017), PID control (Gregory et al., 2020), the piston pump (Madukauwa-David et al., 2020), etc. MCL with coronary circulation can provide trainings for coronary surgeries (Park et al., 2006; Park et al., 2007) as well as investigate the interaction between medical devices and coronary arteries (Calderan et al., 2016; Madukauwa-David et al., 2020). In order to more comprehensively study the effects of medical devices on various circulations, some more complex MCLs were built (Rezaienia et al., 2016; Rezaienia et al., 2017; Gregory et al., 2020). For example, the rotary blood pump (RBP) installed in the descending aorta can increase the CO, but the pump has an influence on the blood perfusion of the heart, brain and kidneys. Hence, MCL with coronary, cerebral, renal circulation was developed by Rezaienia et al. to study the hemodynamic responses of the CVS under the condition of RBP operation (Rezaienia et al., 2016; Rezaienia et al., 2017). Additionally, the forearm circulation were incorporated to the MCL for the arteriovenous fistula eligibility system testing (Loree et al., 2015) and the medical palpation training (Jeong et al., 2020). It can be noted that the inclusion of multiple circulations in the MCL expands the capability of the MCL. Moreover, MCL with multiple circulations is gradually becoming a trend and requirement for MCL construction with the purpose of testing newly developed CADs, such as the Aortix (Procyrion Inc., TX, United States) for increasing CO and renal perfusion (Annamalai et al., 2018; Anouti and Gray, 2019).

## 5 Discussion

After decades of development, MCL has proven to be a significant and necessary platform for conducting *in vitro* tests of CAD, studying the hemodynamics in the organs and performing surgical trainings. To meet the requirements of different applications, the MCL is usually to be modularized, and components of the MCL could be flexibly and easily retrofitted. As an important application, CAD *in vitro* evaluation requires MCL to accommodate the target CAD or various types of CADs. The notable features of MCL for different CADs are listed in Table 2. For studying the PHV in MCL, the corresponding valve should be replaced. The visualization window is normally designed for a better observation of the PHV motion. For testing TAH, the ventricle and atrium chambers of MCL should be replaced by the TAH itself. The bypass from ventricle to artery in the MCL is necessary for the implantation of LAVD, RVAD or BiVAD. The simulated percutaneous access is needed in the MCL for inserting pVAD and IABP. On the one hand, the difference in the MCL structure lies in the different interventions or positions of the CADs to be tested, such as the structure difference in the MCLs testing different types of VADs. On the other hand, due to different focuses on studying CADs, the functional modules and measurement methods are different in the MCLs, for example, the PHV motion is an important issue so that the visualization window is necessary.

**TABLE 2:** The features of the MCL for the different types of CADs.

CAD types	Notable features
PHV	Replacement of the PHV for the corresponding valve
	Visualization window
TAH	Replacement of TAH for the ventricle and atrium chambers
	Both systemic and pulmonary circulatory loops
LVAD, RVAD, BiVAD	Bypass from the ventricle to the artery
pVAD, IABP	Percutaneous access from aorta to the ventricle

As presented in Section 1, the MCL simulates the major functions of the human CVS through electronic, mechanical and hydraulic components. The organs or parameters to be simulated include LV, LA, RV, RA, SAC, PAC, SVC, PVC, SVR, and PVR. Thereinto, the left and right ventricles can be simulated by a chamber driven by a pump, a linear motor or a pneumatic system. The compliance can be realized by a container with gas storage above and liquid storage below. The peripheral resistance is generally achieved by resistance valves. Generally, these components belong to the systemic and pulmonary circulations. These components can be integrated freely to build different circulatory loops for diverse tests. An MCL with a single circulatory loop can be simply built and used for the basic CAD test. However, when testing specific CADs, there are some indispensable components in the MCL system which has been documented in Table 3. The MCL for testing PHV in left side of the heart, the LVAD and the IABP must contain LV, LA, SAC, SVC, SVR. In terms of CAD for right side of the heart, the MCL must contain RV, RA, PAC, PVC, and PVR. BiVAD requires MCL to have both systemic and pulmonary circulatory components. Since TAH replaces the human ventricles and valves, the functions of LV and RV are not necessary in the MCL, but the other components used to achieve the systemic and pulmonary circulation are required.

**TABLE 3:** The necessary elements in the MCL used for testing CADs.

	PHV		TAH	VAD			IABP
	Left side of the heart	Right side of the heart		LVAD	RAVD	BiVAD	
LV	√	—	—	√	—	√	√
LA	√	—	√	√	—	√	√
RV	—	√	—	—	√	√	—
RA	—	√	√	—	√	√	—
SAC	√	—	√	√	—	√	√
PAC	—	√	√	—	√	√	—
SVC	√	—	√	√	—	√	√
PVC	—	√	√	—	√	√	—
SVR	√	—	√	√	—	√	√
PVR	—	√	√	—	√	√	—

**FIGURE 4:**
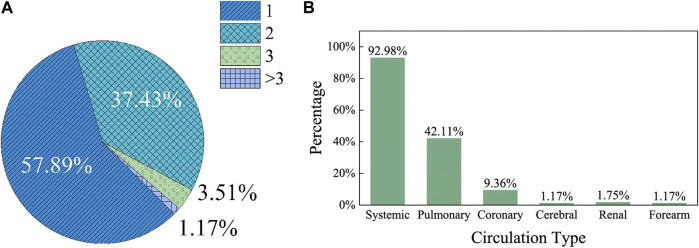
The number and types of circulations counted in the MCLs statistically from the references. **(A)** The circulations count in a single MCL presented in a single literature; **(B)** The circulation types count in a single MCL presented in a single literature.

With a single circulation and necessary elements, MCL is able to fulfill the basic requirement to test a given CAD. However, as CADs are usually integrated to CVS, their effects are normally not limited to a single circulation. Therefore, more comprehensive evaluation of the effect of CADs on the CVS would be achieved by introducing an MCL of multi-circulatory loops, which could include systemic circulation, pulmonary circulation, coronary circulation, cerebral circulation, renal circulation and forearm circulation. Among the MCL-related references of this article, 57.89% of the MCLs have only one circulation, while 37.43% of them have two circulations, and only 5% of them have three or more circulations as shown in Figure 4A. Figure 4B presents the types of circulations in the MCLs in detail. 92.98% of the MCLs presented in the literatures contain systemic circulation, while 42.11% of them contain pulmonary circulation, and about 14% of the MCLs have other circulations. This is clearly indicated that most MCLs have only a single circulation, and they are probably the systemic circulation. With the advancement of clinical applications, there is a growing interest in the more comprehensive impact of CADs on the entire CVS. This poses a challenge for studies with multiple circulations and would be a direction of MCL development.

**FIGURE 5:**
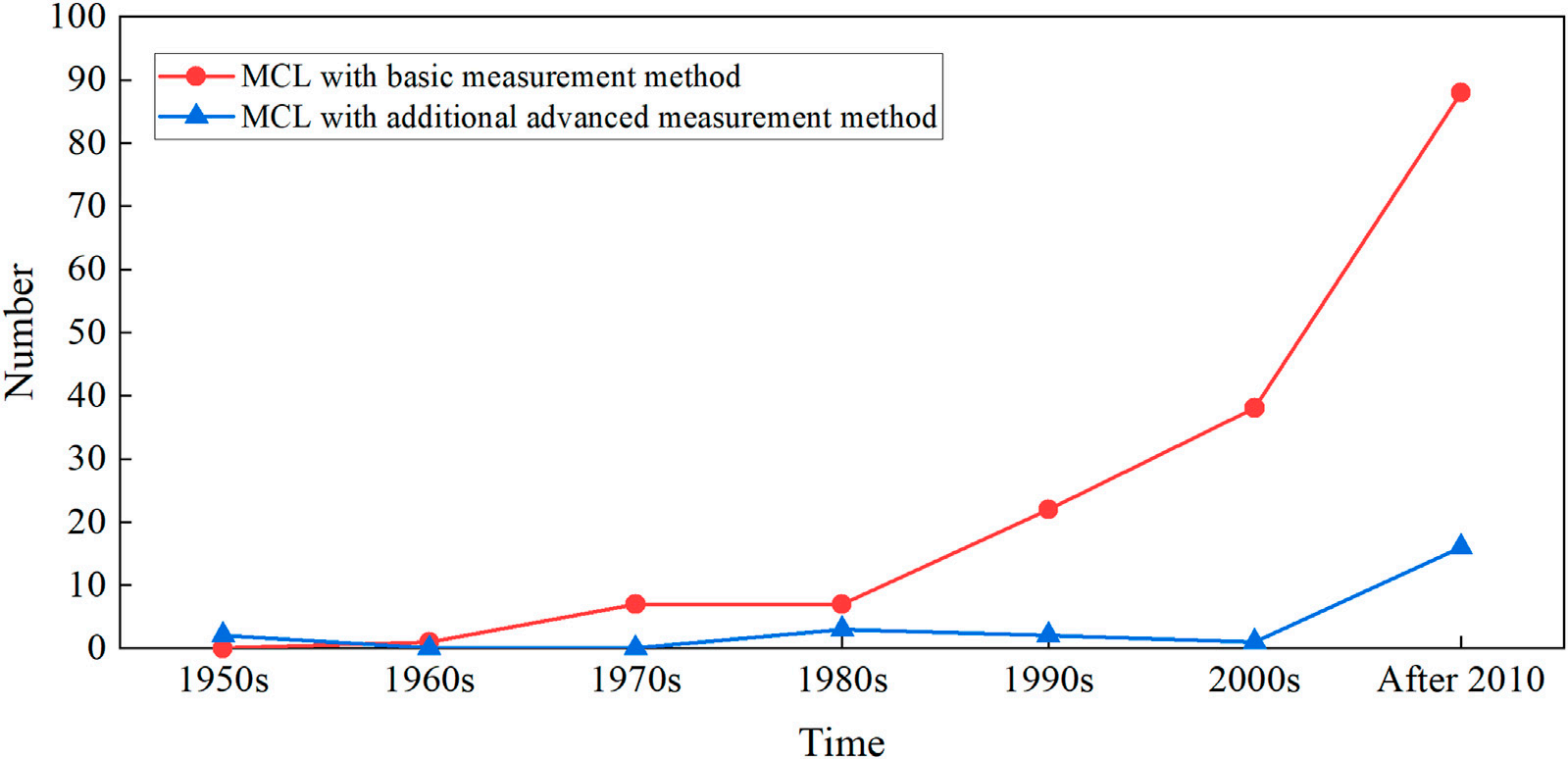
The number of the MCL and its measurement method changes over time, according to the references of this article statistically. The basic measurement method includes pressure, flow measured from the transducers. The advanced measurement method includes PIV, high-speed camera, MRI, etc.

Simulating CVS is the most important requirement of designing MCL, while applying effective observation and measurement methods to MCL is another concern. During *in vitro* experiments, flow parameters of pressure and flowrate will be monitored, since these are the physical quantities that directly reflect the working state of CADs. In addition to some specific transducers and probes for the basic measurement, there are various other advanced methods combined with MCL to test CADs, such as cine cameras (McMillan et al., 1952; Dávila et al., 1956; Heiliger et al., 1987) used in the 1950s to observe valve motion, high-speed cameras (Lin et al., 2000; Linde et al., 2012; Suzuki et al., 2012; Schlöglhofer et al., 2013; Tsuboko et al., 2016; Feng et al., 2017) used since 2000s, PIV (Tanné et al., 2010; Pelletier et al., 2015; Nguyen et al., 2017; Nguyen et al., 2019; Bonfanti et al., 2020; Madukauwa-David et al., 2020) and the MRI (Hattori et al., 2021) used in the last decades to study the flow fields inside the organ phantoms of MCL. These advanced measurement methods make it possible to investigate the internal flow field deeply and comprehensively. Figure 5 counts the measurement methods applied in the references of this article. Almost all studies performed the basic measurement on the flowrate or pressure of MCL, and the number of the investigations rapidly increased in the past 40 years. Among them, there are very limited researches that also used advanced methods. However, the number of those studies significantly increased in the last decade. We believe that more MCL-related studies will be applied in a wider range of applications in the future, and more studies with advanced measurement methods will quickly increase.

Last but not least, durability test is necessary for CAD evaluation, which requires MCL to work under a certain operating condition with CADs for a long period of time. The duration might be few days or weeks for short-term assistance, or might be few years for long-term assistance dependent on clinical needs. Thus, the reliability and durability of MCL become a consideration in that case. The structural design, stability, robustness, seal and fatigue properties of mechanical and electrical components of the MCL are highly concerned. Although, there are few articles available to describe MCLs for CAD durability tests, it is believed that this issue would bring to the attention.

## 6 Conclusion

This article reviews applications of MCL for testing different CADs, including VAD, PHV, TAH, and IABP. The typical structure of MCL used to study these CADs is introduced. Also, MCLs with advanced configurations for special applications, such as the pediatric MCLs are introduced. MCL does not only simulate the circulations but also surgical procedures. Because of widespread applications of MCL, its components should be flexibly modularized and adjusted for testing different types of CADs. Moreover, more advanced measurement methods can be combined with MCLs for better understanding hemodynamics. It is believed that the achievements about MCL and its applications will become abundant and more insightful.
